# 

**Published:** 1914-10-31

**Authors:** 


### CONTENTS.

PAGE

**Bones of Contention in Institution Discipline** 99

**Mental Institutions and the War...** ... ... ... 100

**Hospital and Institutional News ...** ... ... 101

The Cumberland Infirmary, Carlisle—Duty-free Rectified Spirit in Hospitals—The Voluntary Hospitals' Case—An Assistant-Superintendentship Going Begging—Re-opening of the Out-patient Department at Cardiff—New Tuberculosis Dispensary for the City—New Features of the Card-Index System — Collections not Affected by the War Funds—Infirmaries, Prisoners, and Sick Refugees—The Open Door for Naval Orphans—What is the Value of Gifts in Kind?—How Children can Help our Hospitals — Drug Cultivation in Hospital Gardens—The Report on Proprietary Medicines—The Efficiency of the Voluntary System —The Late Mr. Howard Collins—Universities and the Shortage of Medical Students—A Delicate Question of Discipline—A Hedge of Petty Restrictions—The Early-closing Day at Chemists' Shops—Radiologist at Crumpsall Poor-Law Infirmary—An English Doctor with the German Wounded—The Lack of Mental Hospital Beds—The Salaries of Mental Hospital Staffs—Infirmaries and Schemes of Work —A New Tariff for Drugs and Appliances—This Week's Drug Market—To Our Readers.

**The Royal Infirmary, Newcastle-on-Tyne ...** 107

The Special Wards for Wounded Soldiers

Hints for Prospective Followers of the Newcastle Plan.

The Specification for the Newcastle Temporary Wards.

PAGE

**Hospitals and the War** ... ... ... ... 109

Miller Hospital, Greenwich—Bath: Phrase-cards and French-speaking—Belgians at Bristol —Cardiff: 255 more Cases—Flag Day in South Wales—Ipswich Hospital and its War Workers —Leicester: Ambulance Transport—Lincoln: Arrivals from Ostend—Monmouthshire: St. John Ambulance Scheme—Netley: The Welsh Hospital—Sheffield: More Beds and Ambulances Required—Torquay: The Accommodation Question—Tunbridge Wells: Shell Wounds from Antwerp.

**Reports on Hospitals of the United Kingdom—** 113

By SIR HENRY BURDETT, K.C.B., K.C.V.O.

Poor-Law Infirmaries and Union Hospitals.

**The Editor's Letter-Box** ... ... ... ... 115

The National Medical Union and the £90,000 to Panel Doctors—A Critical View of the King's Fund—The Recreations of Convalescing Soldiers.

**Motor-Cars as Ambulance Wagons** ... ... 116

**Round the World** ... ... ... ... ... 117

Fellow-Workers and the Roll of Honour.

**The Profession of Medicine** ... ... ... 118

Panel Doctors and Panel Chemists.

**London Panel Committee** ... ... ... ... 120

The Unallotted Fund and Excessive Prescribing.

**How to Organise Alexandra Day...** ... ... 120

**The Institutional Worker ...** ... (Suppl.) 1

Electrical Cooking for Hospitals.

